# The impact of workplace relationships on nurse-reported quality of care and patient safety in the North West Province

**DOI:** 10.1371/journal.pone.0323620

**Published:** 2025-05-21

**Authors:** Naledi Tlhako, Siedine Knobloch Coetzee, Olateju Jumoke Ajanaku, Erika Fourie

**Affiliations:** 1 NuMIQ Research Focus Area, School of Nursing Science, North-West University, Private Bag, Potchefstroom, South Africa; 2 NuMIQ Research Focus Area, School of Nursing Science, North-West University, Private Bag, Potchefstroom, South AfricaAfrica; 3 NuMIQ Research Focus Area, School of Nursing Science, North-West University, Private Bag, Potchefstroom, South AfricaAfrica; 4 Unit for Business, Mathematics and Informatics, North-West University, Potchefstroom, South Africa; Bangladesh Open University, BANGLADESH

## Abstract

**Background:**

There is a well-established relationship between the quality of workplace interactions among nurses, physicians, their managers, and nurse colleagues, and the subsequent improvement in patient outcomes. However, despite the growing body of research on this topic in developed countries, there remains a notable paucity of literature exploring these dynamics in developing countries.

**Aim:**

To investigate the impact of workplace relationships on nurse-reported quality of care and patient safety.

**Design:**

This study applied a cross-sectional survey design

**Method:**

A multiphase sampling method was applied. Purposive sampling was used to select the province, health sector and hospitals (n = 3). All-inclusive sampling was applied to in-patient units of the selected hospitals and nursing staff in those units (n = 236). Data was collected in April 2021, using validated instruments.

**Results:**

Nurse manager ability, leadership, and support was not experienced as positively contributing to the practice environment and had the most impact on quality of care and patient safety. Collegial nurse-physician relationships were experienced as contributing positively to the practice environment, and had the most impact on adverse events, namely medication errors, patient falls after admission and healthcare-associated infections. Increased exposure to COVID-19 patients resulted in more positive perceptions of nurses regarding collegial nurse-physician relationships. The most common perpetrators of workplace violence were supervisors/managers, followed by nursing colleagues. On average participants experience more personal workplace violence than physical workplace violence. Personal workplace violence had more effect on quality of care, patient safety, and adverse events than physical workplace violence.

**Conclusion:**

Positive workplace relationships or collegiality, especially nurse-manager relationships, appear to have the most impact on nurse-perceived quality of care and patient safety, followed by nurse-physician relationships, and then workplace violence.

**Relevance to clinical practice:**

The focus of education interventions should be on developing leadership, and recruiting and retaining relationship-focused leaders since leaders have the greatest impact on nurse-reported quality of care and patient safety.

## Introduction

Workplace relationships are distinct types of interpersonal relationships that have significant effects on both the people involved and the organizations in which they are formed [1]. Relationships in the workplace serve a variety of purposes, such as task assistance, career advancement, and emotional support [2]. This study focuses specifically on workplace relationships in terms of nurse-physician, nurse- manager and nurse-to-nurse workplace relationships and will focus on both positive workplace relationships or collegiality, as well as negative workplace relationships or workplace violence (WPV). Positive workplace relationships foster collaboration, trust, open communication, mutual support and innovation, creating a safe and enjoyable environment [3]. A negative workplace relationship involves threats, abuse or assault that jeopardize an employee’s health or wellbeing and safety, including during commuting [4]. Decades of research on nurse‐physician workplace relationships showed that the main facilitators and barriers influencing the relationships included: (1) communication and collaboration; (2) roles and responsibilities; (3) hierarchy and education; and (4) liability [5,6]. Positive relationships between nurses and physicians are associated with improved nurse-perceived patient safety and quality of care [6–8], with better communication and collaboration between the two professions resulting in improved patient and nurse outcomes, a positive practice environment, and decreased cost of healthcare [9–11].

Nurse management, leadership, and support of nurses are highly correlated with nurse outcomes, perceptions of the practice environment [12], quality of care and patient safety [13], as well as nurse and patient satisfaction [14]. The nurse managers have a direct or indirect impact on the quality of care and patient safety. A direct impact is through outcomes such as staff satisfaction with job factors, staff health and wellbeing, and processes such as staff’s relationships with work, their colleagues, and the organization [12]. An indirect association of leadership with quality of care and patient safety was found in patient mortality ([13,15], complications of immobility [16], medication errors [17], patient falls [15], pressure ulcers [18], hospital-acquired infections [15]) and length of hospital stay [17].

It is well documented that hospital environments are often plagued by vertical and horizontal workplace violence in the form of physical and psychological abuse, which disrupts collegial workplace relationships and healthcare delivery [19]. Vertical violence is a pattern of behavior that is usually directed against subordinates and is directed at someone who occupies a position at a lower or higher level of the hierarchical scale. Several studies have identified nurse managers and senior staff members [20], as well as physicians [21] as perpetrators of workplace violence. In the 2019–2020 American Nurses Association (ANA) and Healthy Nurse, Healthy Nation (HNHN) Survey, 23% of nurses reported having encountered vertical aggression or bullying from people in positions of authority [22]. A pattern of aggressive behaviors, such as bullying and intimidation, between nurses who are at the same rank level in the hierarchy is known as horizontal violence [23]. In South Korea, 82% of nurses indicated being exposed to some form of horizontal violence [24], while in Cape Town, South Africa, 44% of nurses working in public hospitals had experienced horizontal violence [25].

Workplace violence includes physical and psychological abuse. Physical workplace violence involves using force such as shoving, biting, pinching, shooting, kicking, slapping, stabbing, or sexual assault, regardless of injury [26]. Psychological abuse is defined as using offensive language or words that disrespect an individual’s dignity and worth, leading to abusive, humiliating, intimidating, and insulting behaviors [26]. Furthermore, Khoshknab et al. [27] are of the view that although physical and psychological violence is common, psychological violence occurs more frequently. According to a recent review by Mobaraki et al. [28], the most prevalent forms of workplace violence are, in order of prevalence from most to least common: verbal, physical and psychological.

A substantial amount of research has been done concerning workplace violence and its effect on the nurse, but less is known about its eroding effect on quality of care and patient safety [24]. Internationally workplace violence have been linked to compromised quality of care and patient safety [29] resulting in adverse events such as hospital acquired infections [30] patient mortality [31], medication errors [32], patient falls and pressure ulcers [33]. However, there is a dearth of such literature in developing countries.

A systematic review [34] revealed that 62.3% of nurses experienced workplace violence in Africa, with recent studies done in Botswana [35], Nigeria [36], Ethiopia [37] and Ghana [38] all indicating poor nurse-physician collaboration within developing countries leading to a breach in patient safety [38]. Although recent studies in South Africa concluded that patient and families were the main perpetrators of workplace violence in emergency units [39] and psychiatric hospitals [40] but in the private healthcare sector horizontal workplace violence was the most common [41].

Although much is published on the effect of workplace violence on the nurse, less is known about the effect on quality of care and patient safety, especially in the African context [20,42]. Therefore, this study will focus on how workplace relationships influence the quality of care and patient safety as well as the frequency of work-related, personal and physical workplace violence between nurses and physicians, their managers and nursing colleagues.

## Methods

South Africa has a dual healthcare system, namely a public sector that provides care to approximately 83% of the population of the country, and a private sector that provides care to the rest of the population [43]. The study was conducted in the public healthcare sector of the North West Province (NWP) of South Africa. A cross-sectional survey design was applied in this study, using a valid and reliable self-administered, paper-based survey that measures five variables, namely the Practice Environment Scale of the Nurse Work Index (PES-NWI), Negative Acts Question Revised (NAQ-R), quality of care, patient safety, and adverse events, and selected nurse and unit demographics.

### Positive workplace relationships or collegiality

The PES-NWI was used to measure positive workplace relationships or collegiality regarding the nurse-physician relationship and the nurse-manager relationship. The following two subscales were used: nurse manager ability, leadership, and support of nurses; and collegial nurse-physician relations. This scale consists of 11 questions answered on a Likert scale from 1 to 4, where 1 represents strongly disagree and 4 strongly agree; a mean score of 2.5 and more indicates a positive practice environment. Scores on the PES-NWI are valid and reliable for measuring the nursing practice environment across samples in the United States (US) and non-US countries [44–46] with the subscales having a Cronbach’s alpha of 0.88 (Nurse participation in hospital affairs); 0.84 (Nursing foundations for quality of care); 0.84 (Nurse manager ability, leadership, and support of nurses); 0.75 (Staffing and resource adequacy); and 0.89 (Collegial nurse-physician relationships) respectively.

### Workplace violence

Workplace violence was calculated using the NAQ-R (Negative Acts Questionnaire–Revised). There are three subscales that make up the NAQ: work-related, personal, and physical workplace violence. In this study, only the personal and physical workplace violence subscales were included. These comprised 15 questions, with responses on a Likert scale of 1–5, with 1 being never and 5 denoting daily. Subscale items are calculated and averaged to determine subscale scores. The test exhibits strong validity and reliability [47] with Cronbach’s alpha for subscales being: Physically Intimidating bullying: α = 0.83; Person-related bullying: α = 0.96.

### Quality of care

Four questions that were utilised as single items were used to assess the nurses’ perceptions of the quality of the care. These have been utilised in multi-country studies in Asia [48], Europe [49], North America [50], South Africa [51], and other regions. They were also included in multiple systematic reviews and meta-analyses [52]. Questions were answered according to a Likert scale ranging from 1 representing frequently to 4 representing never.

### Patient safety

In this study, two questions measuring nurse-perceived patient safety were used. These questions have been used as single items in multi-country studies conducted in North America [50], Asia [48], South Africa [51], and Europe [49]. They have also been included in numerous systematic reviews and meta-analyses. These questions were answered according to a Likert scale of 1–5, where 1 is good and 5 is failing. The remaining 8 questions came from surveys on patient safety (SOPS) culture conducted by the Agency for Healthcare Research and Quality (AHRQ). The responses were given on a Likert scale of 1–5, with 1 denoting strong agreement and 5 denoting strong disagreement.

### Adverse events

In multi-country studies conducted in Europe [49], North America [50], Asia [48], and South Africa [51], the nurse-perceived adverse events questionnaire consisted of seven questions. These questions have also been included in numerous systematic reviews and meta-analyses [52]. These questions were answered according to a Likert scale of 0–5, where 0 equals never and 5 equals frequently.

The study also included certain individual and unit variables, including age, gender, employment status, nursing category, Bachelor of Nursing degree, specialty of the current unit, nursing clinical specialty, level of clinical specialty, years worked as a nurse, and years employed in the hospital.

### Population and sample

A multiphase sampling technique was used as the overall larger study applied and all-inclusive sampling of the Provinces in South Africa, but NWP was purposively chosen for this study, as was the public sector. Purposive sampling was also applied to select the hospitals in the public sector, including the regional and district hospitals (n = 3) neighboring the largest tertiary hospital in the province. There is no central hospital in the NWP. When choosing the study’s units and participants, all-inclusive sampling was used. The study covered every in-patient setting, including the emergency rooms and theatres. The participants included community service nurses, enrolled nurses, auxiliary nurses, registered nurses, and midwives. Nurses who have worked in the chosen hospital for less than three months may not be able to provide an overall assessment of factors such as the working environment, quality of care, or patient safety within their units or even hospitals since they are still new employees, this was used as an exclusion criterion. Participants were recruited through the researcher presenting the research project to eligible participants in every in-patient unit to both day and night shift, whereafter they had 24 hours to choose whether to participate or not. The distribution of 319 (N) surveys at the tertiary hospital resulted in 123 (N) returned surveys; distribution of 146 (N) surveys at the regional hospital resulted in 72 (N) returned surveys; and distribution of 76 (N) surveys at the small district hospital resulted in 41 (N) returned surveys. There was a total of n = 236 nurses in the overall sample. For the class of models like the ones used in this study [53] sample size of 200 provides sufficient statistical power to conduct the exploratory and confirmatory factor analysis and structural equation modelling. Statistical estimation involving a sample of 200 typically results in stable estimates of selected fit indices used to determine the degree of fit between the pattern of relationships in the data and those proposed in a model [54].

### Data collection

Data was collected from 11–23 April 2021. After ethical approval was obtained from the North-West University Health Research Ethics Committee (NWU-HREC) and the NWP Department of Health (DoH). After receiving goodwill consent from the respective Chief Executive Officers, they served as gatekeepers and referred the researcher to the nursing service managers of the facilities, who acted as mediators for the study. The nursing service managers had to select an independent person to assist with data collection. When no independent person could be appointed, the principal investigator employed a research assistant for this purpose.

The researcher explained the project to potential participants in each of the units of the selected hospitals, in the presence of an independent person. The independent person obtained informed consent from possible participants after a minimum of 24 hours allowing the participants adequate time to make an informed decision on their willingness to participate.

Afterwards participants were provided with a survey, an NWU-branded pen, a sachet of coffee and a leaflet for entry into a lucky draw. The participant had 2 days to complete the survey, at a time and place that was convenient for them. After participants completed the survey, they sealed it in a C4 envelope provided and placed it in a box located in the mediator’s office. The researcher collected the boxes after one week.

### Data analysis

Data was analysed using the computer software program SPSS version 27 [55]. Descriptive statistics were used to present the demographic data of the participants. Confirmatory factor analysis, and Cronbach’s alpha were conducted to ensure the validity and reliability of the scales. Factor scores were derived by calculating the means of the questions within each factor. The correlations between workplace relationships, quality of care, patient safety, and adverse events were conducted using Spearman’s rank-order correlations. Independent t-tests, ANOVAs, and Spearman’s rank-order correlations were conducted to determine the association between participant demographics and workplace relationships. Cohen’s d effect sizes were used to determine the importance of the differences in means, where 0.2 is considered as small, 0.5 as medium, and 0.8 as large. The magnitude of correlations is regarded as the effect size, where 0.1 is considered as small, 0.3 as medium and 0.5 as large. The effect size is independent of sample size and is a measure of practical significance.

### Ethics

This study received ethical approval from the NWU-HREC (NWU-000270–21-A1), and the NWP DoH, with goodwill consent from the three participating hospitals. The ethical considerations of the study adhere to the principles of ethics as stipulated by the DoH guidelines on ethics in health research [56]. Participants were informed that participation in this study is voluntary, and they may withdraw from the study at any time. Participant confidentiality was ensured through participants completing the surveys anonymously, with no direct or indirect identifiers, e.g., date of birth or unique personal characteristics included in the survey. Unique codes, known only by the researcher were used to code surveys to enable data analysis at hospital and unit level.

## Results

### Demographic details of nurse participants and units

A total of 542 surveys were distributed, of which 236 were returned, with a response rate of 43.5%. The total number of participants across all sectors was 236. The majority of the participants were female (n = 193; 81.8%), in the category of registered nurses and/or midwives (n = 135; 57.2%) and working in medical wards (n = 47; 20.0%). There were 19.5% (n = 46) with a bachelor’s degree in nursing, and 21.6% (n = 51) with specialty training, mostly at the level of a postgraduate diploma/degree (n = 49; 20.7%). Most of the nurses were permanently employed (n = 202; 86%). The majority had occasional (n = 71; 30%) and routine contact with COVID-19 patients (n = 86; 36.4%). The most common perpetrators of workplace violence were supervisors/managers (n = 79; 33.4%), followed by nursing colleagues (n = 62; 26.2%). On average the nurses were 40.4 years of age (SD 10.1), had worked as a nurse in any category for an average of 12.6 years (SD 10.0), and had spent 9.5 years (SD 8.9) in the specific hospital (see Table 1).

**TABLE 1:** Demographics (n = 236).

Demographics			
**Frequencies**			
**Variable**		**n**	**%**
Gender	Female	193	81.8
	Male	43	18.2
Nurse Category	RN/M	135	57.2
	CSN	7	3.0
	EN/M	31	13.1
	AN	62	26.3
Specialty area of current unit	Medical	47	20.0
	Surgical	21	8.9
	Trauma	24	10.2
	Maternity	37	15.7
	Critical care/ Intensive Care Units	27	11.4
	Theatre	14	6
	Paediatrics	20	8.5
	Other	45	19.1
Bachelor Degree in Nursing	Yes	46	19.5
	No	188	79.6
Specialty training in nursing	Yes	51	21.6
	No	182	77.1
If yes, at what level is the specialty training?	Certificate	25	10.6
	Post-graduate diploma	49	20.7
	Masters	1	0.42
Employment Status	Permanently employed (full-time)	202	86.0
	Permanently employed (part-time)	8	3.4
	Temporary/Agency	25	10.6
Hospitals	Public hospital 1	123	52.1
	Public hospital 2	72	30.5
	Public hospital 3	41	17.4
**Covid 19**			
**Variable**		**n**	**%**
How often do/did you have to directly care for COVID-19 patients in your practice setting?	Never	29	12.3
	Rarely	38	16.1
	Occasionally	71	30
	Routinely	86	36.4
**Workplace Violence**			
**Variable**		**n**	**%**
Perpetrators of workplace violence	Physicians	10	4.2
	Supervisors/managers	79	33.4
	Nursing colleagues	62	26.2
**Descriptive**			
**Variable**	**n**	**M**	**SD**
Age	225	40.4	10.1
Years worked as nurse	224	12.6	10.0
Years worked in current hospital	200	9.5	8.9

### Descriptive statistics

The descriptive statistics and the reliability of the instruments used are shown below in Table 2. The subscales of the NAQ-R and PES-NWI were considered reliable. Only selected items of the AHRQ were included, and therefore an exploratory factor analysis was conducted. The pattern matrix is divided into two subscales with regard to the selected items of the AHRQ survey: 1) communication (We discuss ways to prevent errors from happening again; Regularly review work processes to determine if changes are needed to improve patient safety; The actions of the hospital management show that patient safety is a top priority; Staff feels free to question the decisions or actions of those in authority; Staff speak up if they see something that may negatively impact patient care); and 2) staffing and response to errors (Staff feel that their mistakes are held against them; There is a lack of support for staff involved in patient safety errors; Relies too much on temporary, float or agency staff), with a Kaiser-Meyer-Olkin measure of sampling adequacy of 0.729, and 49.89% of the total variance explained. However, the staffing and response to errors were not reliable and were thus reported as single items.

**Table 2:** Overview of study characteristics of the included studies.

Workplace Relationships				
**Workplace violence variables**	**Mean (M)**	**Ranges**	**Standard Deviation (SD)**	**Cronbach’s Alpha**
Personal workplace violence	1.61	1–5	0.80	0.93
Physical workplace violence	1.51	1–5	0.88	0.81
**Practice environment variables**	**Mean (M)**	**Ranges**	**Standard Deviation (SD)**	**Cronbach’s Alpha**
PES-NWI: Nurse manager ability, leadership and support of nurses	2.45	1–4	0.79	0.85
PES-NWI: Collegial nurse-physician relations	2.86	1–4	0.71	0.90
**Quality of Care**				
**Variable**	**Mean (M)**	**Ranges**	**Standard Deviation (SD)**	**Cronbach’s Alpha**
In general, how would you describe the quality of nursing care delivered to patients in your work setting?	2.12	1–4	0.78	Single item
Would you recommend where you work to your family and friends needing healthcare?	2.02	1–4	0.96	Single item
How confident are you that your patients and their caregivers can manage their care after discharge?	2.52	1–4	0.88	Single item
**Patient Safety**				
**Variable**	**Mean (M)**	**Ranges**	**Standard Deviation (SD)**	**Cronbach’s Alpha**
Communication errors	2.34	1–5	0.85	0.75
Please give your current practice setting an overall grade on patient safety.	2.23	1–5	1.01	Single item
How sure are you that management will act to resolve problems in patient care that nurses identify?	2.59	1–4	0.99	Single item
Relies too much on temporary, float or agency staff	3.28	1–5	1.42	Single item
Staff feel like their mistakes are held against them	2.53	1–5	1.27	Single item
There is a lack of support for staff involved in patient safety errors	2.71	1–5	1.32	Single item
**Adverse Events**				
**How often would you say the following occurs involving you or your patients**	**Mean (M)**	**Ranges**	**Standard Deviation (SD)**	**Cronbach’s Alpha**
Medication errors	1.80	1–5	1.01	Single item
Patient fall with injury after admission	1.45	1–5	0.72	Single item
Healthcare-associated infections	1.92	1–5	0.96	Single item
Treatments/ procedures resulting in unintended harm	1.66	1–5	0.84	Single item

On average, participants experience more personal workplace violence (M=1.61; SD 0.80) than physical workplace violence (M=1.51; SD 0.88). Regarding the PES-NWI, nurse manager ability, leadership, and support of nurses (M=2.45; SD 0.79) is below the mean of 2.5, indicating that it is not experienced as contributing towards a positive practice environment, although collegial nurse-physician relationships were experienced positively (M=2.86; SD 0.71). Regarding quality of care, nurses were only somewhat confident that patients and their caregivers could manage their care after discharge (M=2.52; SD 0.88), while other items such as quality of care delivered to patients (M=2.12; SD 0.78) and recommending your workplace to family and friends needing healthcare (M= 2.02; SD 0.96) were experienced positively. Patient safety issues included nurses being only somewhat confident that management would act to resolve problems in patient care that nurses identified (M=2.59; SD 0.99) and being neutral with regard to relying too much on temporary, float, or agency staff (M=3.28; SD 1.42), staff feeling that their mistakes are held against them (M=2.53; SD 1.27), and lack of support for staff involved in patient safety errors (M=2.71; SD 1.32). Overall grades on patient safety (M=2.23; SD 1.01) and the communication subscales (M=2.34; SD 0.85) were experienced positively. With regard to adverse events, healthcare-associated infections occurred most often (M=1.92; SD 0.96), followed by medication errors (M=1.80; SD 1.01). Patient falls after admission (M=1.45; SD 0.72) and treatment procedures resulting in unintended harm (M=1.66; SD 0.84) were less frequent.

### Correlations between main study variables

In Table 3 (see below) the correlations between the main study variables are described. The PES-NWI: Nurse manager ability, leadership, and support of nurses had the most effect on quality of care and patient safety, with mostly medium to large, statistically significant negative correlations, especially the item management will act to resolve problems in patient care (r = -0.498; p = 0.000) and communication errors (r = -0.467; p = 0.000). It also had small to medium, statistically significant positive correlations with staff feeling that their mistakes are held against them (r = 0.320; p = 0.000), and relying too much on temporary, float, or agency staff (r = 0.138; p = 0.000). The PES-NWI: Collegial nurse-physician relationships had the most impact on adverse events, especially medication errors (r = -0.303; p = 0.000) and healthcare-associated infections (r = -0.299; p = 0.000), with medium, statistically significant negative correlations, and likewise with patient falls after admission (r = -0.244; p = 0.000).

**Table 3:** Correlations between study variables.

		Quality of care			Patient safety						Adverse events			
	Values	Quality of nursing care delivered to patients in your work setting	Would you recommend where you work to your family and friends needing healthcare?	Confident that patients and their care-givers can manage their care after discharge	Communication errors	Overall grade on patient safety	Management will act to resolve problems in patient care	Relies too much on temporary, float or agency staff	Staff feel that their mistakes are held against them	Lack of support for staff involved in patient safety errors	Medication errors	Patient falls after admission	Health-care-associated infections	Treatment procedures resulting in unintended harm
**PES-NWI: Nurse manager, ability, leader- ship and support of nurses**	**r**	-0.348^**^	-0.385^**^	-0.288^**^	-0.467^**^	-0.355^**^	-0.498^**^	0.138^*^	0.320^**^	-0.195^**^	-0.164^*^	-0,060	-0.156^*^	-0,058
	**p**	0.000	0.000	0.000	0.000	0.000	0.000	0.042	0.000	0.004	0.017	0.385	0.024	0.396
**PES-NWI: Collegial nurse- physician relationship**	**r**	-0.371^**^	-0.317^**^	-0.245^**^	-0.274^**^	-0.327^**^	-0.343^**^	0.082	0.160^*^	-0.063	-0.303^**^	-0.244^**^	-0.299^**^	-0.120
	**p**	0.000	0.000	0.000	0.000	0.000	0.000	0.230	0.018	0.350	0.000	0.000	0.000	0.082
**Personal Workplace violence**	**r**	0.275^**^	0.345^**^	0.191^**^	0.340^**^	0.246^**^	0.212^**^	-0.105	-0.246^**^	0.342^**^	0.305^**^	0.145^*^	0.283^**^	0.190^**^
	**p**	0.000	0.000	0.004	0.000	0.000	0.001	0.116	0.000	0.000	0.000	0.031	0.000	0.005
**Physical Workplace violence**	**r**	0.220^**^	0.257^**^	0.205^**^	0.268^**^	0.186^**^	0.149^*^	-0.121	-0.213^**^	0.203^**^	0.193^**^	0.175^**^	0.288^**^	0.218^**^
	**p**	0.001	0.000	0.002	0.000	0.005	0.025	0.071	0.001	0.003	0.004	0.009	0.000	0.001

** **Correlation is significant at the 0.01 level (two-tailed).**

* **Correlation is significant at the 0.05 level (two-tailed).**

In contrast, personal workplace violence had small to medium, statistically significant positive correlations with most aspects of quality of care and patient safety, especially regarding whether the nurses would recommend where they work to family and friends needing healthcare (r=0.345; p=0.000), lack of support for staff involved in patient safety errors (r=-0.342; p=0.000) and communication errors (r=0.340; p=0.000). This was also the case for adverse events, especially medication errors (r=0.305; p=0.000) and healthcare-associated infections (r=0.283; p=0.000).

Physical workplace violence had mostly small but also a few medium correlations with quality of care and patient safety, especially in terms of communication errors (r=0.268; p=0.000) and whether the nurses would recommend where they work to family and friends needing healthcare (r=0.257; p=0.000). Physical workplace violence also had mostly small with a few medium correlations with adverse events, especially healthcare-associated infections (r=0.288; p=0.000) and treatment procedures resulting in unintended harm (r=0.218; p=0.000).

### Association between demographic profile and workplace relationships

There was a medium association between having specialty training and personal workplace violence (d = 0.66; p = 0.000) and a small association between physical workplace violence (d = 0.37; p = 0.020), with those with specialty training having a higher mean than those without ([Personal workplace violence] M = 2.00; M = 1.50; [Physical workplace violence] M = 1.77; M = 1.44). There was a small association with PES-NWI Leadership, management, and support of nurses (d = 0.32; p = 0.049), with those without specialty training rating that aspect of the practice environment higher (M = 2.51) than those without specialty training (M = 2.26). There was a medium association with PES-NWI: Leadership, management, and support of nurses and nurse category, with enrolled nursing auxiliaries rating that aspect of the practice environment higher than all of the other categories of nurses (d = 0.44–0.50; p = 0.031), with enrolled nursing auxiliaries experiencing this more positively than registered nurses, community service nurses and enrolled nurses and/or midwives (M = 2.72 [enrolled nursing auxiliaries]; M = 2.36 [registered nurses and/or midwives]; M = 2.36 [community service nurses] and M = 2.39 [enrolled nurses). There was a small to medium association between PES-NWI: Collegial nurse-physician relations and routinely caring for COVID-19 patients (d = 0.22–0.62; p = 0.028), in that the more that nurses worked with COVID-19 patients, the more positively they experienced collegial nurse-physician relations. There was no association with gender, having a Bachelor’s degree, hospital where employed, employment status, specialty of the unit, level of specialty training, age, years worked as a nurse or years worked in the specific hospital.

## Discussion

The most common perpetrators of workplace violence were supervisors/managers, followed by nursing colleagues. On average, participants experience more personal workplace violence than physical workplace violence. A higher incidence of workplace violence was associated with specialty training. Personal workplace violence had more effect on quality of care, patient safety, and adverse events than physical workplace violence, both having the most impact on recommending their place of work to family and friends needing healthcare, communication errors, and lack of support for staff involved in patient safety errors. Overall, nurse managers’ ability, leadership, and support were not experienced as positively contributing to the practice environment and had the most effect on quality of care and patient safety, especially with regard to management resolving problems in patient care, communication errors, and recommending their place of work to family and friends needing healthcare. Collegial nurse-physician relationships were experienced as contributing positively to the practice environment, and after nurse managers’ ability, leadership, and support, these had the most effect on quality of care and patient safety, and specifically on adverse events, namely medication errors, patient falls after admission and healthcare-associated infections. The nursing category was linked to perceptions of leadership, management, and support of nurses. Finally, increased exposure to COVID-19 patients resulted in nurses having more positive perceptions of collegial nurse-physician relationships.

Leadership, management, and support of nurses were not perceived to be present in the practice environment; in addition, it was also perceived that managers/supervisors were most frequently the perpetrators of workplace violence. National [25,57]; and international [58,59] studies have similarly found that managers/supervisors are commonly the perpetrators of workplace violence. It is apparent that managers/supervisors influence nurses’ exposure to workplace violence in both direct and indirect ways. Direct factors include using workplace violence as a disciplinary measure, to sustain hierarchical work practices, top-down driven leadership, leadership voids, and lack of interpersonal skills; while indirect factors include the normalization of workplace violence, indifference to ethical issues related to bullying, and/or a lack of managerial abilities to address the phenomenon [60]. A systematic review concluded that when nurse managers practice moral leadership and attentively address nurses’ individual concerns while maintaining their privacy, they are less likely to perpetrate workplace violence and reduce its occurrence. When nurses practice authoritarian leadership the likelihood of workplace violence increases [61]. Furthermore, when levels of stress increase like they did during the pandemic, those with greater perceived power often express more aggression and those who perceive themselves as less powerful often believe that their only option is to submit and abide [22].

This is a problematic finding, as it is also leadership, management, and support of nurses that had the most impact on nurse-perceived quality of care and patient safety. It is well established from systematic reviews on nurse leadership [12,13] that leadership, management, and support of nurses have an impact on nurse, patient, and system outcomes. However, of all of the studies included in the systematic reviews, only two were from Africa [62], and these focused only on nurse outcomes. Authors agree that managers/supervisors have both a direct and indirect impact on patient outcomes: directly due to the fact that they make decisions about human resource variables (e.g., staffing and resources, overtime) that are connected to patient care outcomes [13], and indirectly through the health and wellbeing of nursing staff that impact on work performance (e.g., job satisfaction, turnover intention) [63], as well as through the quality standards of the organization (e.g., consultation and involvement of nurses in decision making, education, and training) [64].

According to the systematic review of Wong et al. [13] leadership directly impacts patient outcomes regarding five aspects: patient satisfaction; patient mortality; adverse events (behaviour problems, restraint use, complications of immobility, fractures, medication errors, patient falls, catheter use, pressure ulcers, inadequate pain management, and hospital-acquired infections); complications; and patient healthcare utilisation. These patient outcomes include patient reports, nurse-perceived assessments, and actual recorded incidents, all of which are considered reliable forms of measuring patient outcomes, especially nurse-perceived assessments [65].

A focus on leadership development, as well as the recruitment and retention of relationally orientated leaders, are therefore crucial to improving patient, nurse, and organizational outcomes. A systematic review on leadership education interventions [12] has shown that although these interventions differ widely with regard to programme content, length, and delivery, most showed significant increases in self-reported leadership and observed leadership practices. However, such interventions must be evidence-based, theoretically grounded, and incorporate cultural, social, and the institution’s contextual factors [66].

Collegial nurse-physician relationships were experienced as contributing positively to the practice environment, and after nurse managers’ ability, leadership, and support, this aspect had the most effect on nurse-perceived quality of care and patient safety, especially on the incidence of adverse events. This is in accordance with the international literature on the topic, where collegial nurse workplace relationships have positive effects on quality of care and patient safety, including patient satisfaction [67], recorded incidents of patient outcomes [10] and nurse-perceived assessments [6–8]. An interesting finding from this study is that increased exposure to COVID-19 patients resulted in nurses having improved perceptions of physician-nurse collaboration. This may point to the fact that there was better collaboration and communication between nurses and physicians when caring for COVID-19 patients, due to the sheer volume and changes in the workplace environment related to the COVID-19 pandemic. This was also confirmed by Matusov et al. [68], who reported increased team interdependence and appreciation among nurses and physicians during the COVID-19 pandemic.

Personal workplace violence was experienced more than physical workplace violence, and this finding is concurrent with those of international studies [69]. In Africa, studies have reported workplace violence rates ranging from as low as 26.7% in Ethiopia [70] to as high as 85% in South Africa [71]. In all cases, the most common type of workplace violence was found to be non-physical violence [58]. Also, personal workplace violence had a greater effect than physical workplace violence on nurse-perceived quality of care, patient safety, and adverse events. Interestingly, workplace violence had the most effect on recommending their place of work to family and friends needing healthcare, communication errors, and lack of support for staff involved in patient safety errors. In a systematic review conducted by Lanctôt and Guay [42], the outcomes of workplace violence include seven categories of consequences: physical, psychological, emotional, work functioning, relationship with patients/quality of care, social/general, and financial stress [42]. Additionally, Lanctôt and Guay [42] found that among the selected articles in their review, only ten of them addressed issues related to relationships with clients or quality of care [72], with most articles indicating a reduction in quality of care and patient safety. Again, workplace violence education interventions are linked to a decrease in workplace violence, due to the fact that identification of incidents is improved, reporting increases, and therefore situations can be addressed and dealt with; it also improves communication, clarification of roles and responsibilities between professionals, and knowledge of how to address workplace violence incidents [73].

Specialization level was linked to an increased perception of workplace violence, and this was also found by international studies [74]. Interestingly, it was not linked to specialty units, as found in other South African studies [75]. Also, perceptions of the practice environment with regard to leadership, management, and support of nurses were linked to nursing categories, with lower-ranked categories experiencing this subscale more positively than higher-ranked categories. It is generally found in regional and national literature that lower-ranked categories rate the practice environment, quality of care, and patient safety more positively than their higher-ranked counterparts [76]. The common argument for this is that education and training bring greater knowledge and awareness of the practice environment, quality of care, and patient safety.

### Limitations

Nurse-nurse relationships were only measured through the presence of workplace violence, and not collegial or positive relationships; this is a limitation considering that in all instances both nurse-physician and nurse-manager collegial or positive relationships had a greater impact on quality of care, patient safety, and adverse events than workplace violence or negative relationships did. There are two main instruments used to measure collegiality: a 72-item survey titled Nurse-Nurse Collaboration Scale (NNCS), with reliability scores of 0.66–0.99 [77], and a 54-item survey titled Colleague Solidarity of Nurses Scale (CSNS), with reliability scores of 0.63–0.80 [78]. Due to the extent of the scales, it was decided not to include these surveys in the study, and the PES-NWI does not dedicate a portion to nurse-nurse collegial relationships. Further study of workplace relationships is necessary, considering their implications for nurses, patients and organisations. There is also a specific need for studies addressing the management of workplace violence episodes, especially violence perpetrated by co-workers or patients, visitors and patient family members. As well as studies that assess the efficacy of associated measures. This study has some limitations, including its reliance on cross-sectional data, which makes it difficult to draw a conclusion on the causal relationship between collegial relationships and care quality, patient safety, and adverse events. The results of this study, which only included hospitals from the NWP of South Africa, cannot be applied to other contexts. In addition, the province’s hospitals were purposefully sampled; nonetheless, despite the sampling’s vast participant pool, it was not a probability sample.

## Conclusion

This study showed that collegial relationships or positive relationships had a greater impact on quality of care, patient safety and adverse events than workplace violence or negative relationships did. The most important collegial relationship was the nurse-manager relationship, as it had the most impact on quality of care and patient safety; this is problematic as quality of care and patient safety were not perceived as being present in the practice environment of the study participants, as well as the fact that nurse managers/supervisors were experienced as being the greatest perpetrators of workplace violence. Nurse-physician relationships had an impact on quality of care, patient safety, and especially adverse events, indicating that improved nurse-physician relationships improved perceptions of quality of care, patient safety, and adverse events. Also, increased care of COVID-19 patients improved nurses’ perceptions of the nurse-physician collegial relationship, indicating increased collaboration and communication between the professions. Personal workplace violence had a greater effect on quality of care, patient safety, and adverse events than physical workplace violence.

### Relevance to clinical practice

It is clear from the findings that urgent attention must be directed towards leadership development, as well as to the recruitment and retention of relationally orientated leaders, as this aspect is perceived negatively in the participants’ current practice environment, even though it is most highly correlated with quality of care and patient safety. It would seem that the best method to improve leadership practice is through leadership education interventions; these should be evidence-based, theoretically grounded, and based on the local culture, social practices, and context, as these show the most improvement in leadership practices. Researchers especially recommend the combination of experiential learning, such as mentorship and coaching, and action learning [12]. Although workplace violence or negative relationships also impacted on quality of care, patient safety, and adverse events, it appeared from the findings that focusing on the development of collegial or positive relationships would have a greater impact not only on patient outcomes but also on nurse- and organisation outcomes.

## ‘What does this paper contribute to the wider global clinical community?’

- Positive workplace relationships or collegiality seemed to have a greater impact on nurse-perceived quality of care and patient safety than workplace violence.
- Of all relationships, nurse-manager’s/supervisors’ relationships had the greatest impact on nurse-perceived quality of care and patient safety.
- Nurses’ perceptions of the nurse-physician relationship improved as their nursing exposure to COVID-19 patients increased.
